# Prevalence of hypertension in Europe: a pooled analysis of 68,047 adults from 27 countries

**DOI:** 10.1016/j.pmedr.2026.103435

**Published:** 2026-02-28

**Authors:** Jozo Grgic, Vanessa Kristina Wazny

**Affiliations:** a Department of Sports Science and Physical Education, The Chinese University of Hong Kong, Hong Kong Special Administrative Region of China; b NUS Academy for Healthy Longevity, Yong Loo Lin School of Medicine, National University of Singapore (NUS), Singapore 117456, Singapore

**Keywords:** Blood pressure, National cohort, Regional differences, Hypertension, Nation-wide survey

## Abstract

**Objective:**

This study analysed the prevalence of self-reported current/previous hypertension diagnosis in Europe.

**Methods:**

We used data from the Survey of Health, Ageing and Retirement in Europe (2021−2022), covering 68,047 adults aged 50+ years across 27 countries. Weighted prevalence estimates were calculated and stratified by gender, age group (50–64, 65–79, ≥80 years), and European regions.

**Results:**

Among individuals aged 50–64 years, men had a higher prevalence of hypertension diagnosis (39.4% vs. 33.5%). In the 65–79 age group, prevalence was comparable between genders (women: 63.1%, men: 62.6%). In the 80+ age group, women had higher prevalence (78.0% vs. 71.8%). Still, this pattern varied across European regions. Central/Eastern and Southern Europe consistently showed higher prevalence of hypertension compared with Northern and Western regions.

**Conclusions:**

The prevalence of hypertension in Europe increases with ageing and differs by gender and region. The burden is greatest in Central/Eastern and Southern Europe, highlighting the need for region-specific strategies.

## 1 Introduction

A recent pooled analysis examined global trends in hypertension prevalence from 1990 to 2019 (NCD Risk Factor Collaboration (NCD-RisC), 2021). This analysis found an overall increase in prevalence, with higher rates among men (34%) than women (32%) (NCD Risk Factor Collaboration (NCD-RisC), 2021). A limitation of the study is that it only included adults aged 30–79 years. Additionally, for some European countries, such as Austria, data were available only for individuals aged 30–64 years and were derived from datasets generated between 1998 and 1999. Finally, not all European countries were represented (e.g., Bulgaria, Cyprus). The Survey of Health, Ageing and Retirement in Europe (SHARE) provides a unique dataset to study these gaps, as it is a large nationally representative cohort encompassing individuals aged 50+ years from different European countries (Börsch-Supan et al., 2013). This study aimed to examine the prevalence of self-reported hypertension diagnosis in Europe using the SHARE cohort data.

## 2 Methods

### 2.1 Study design and population

Data from the ninth wave (2021–2022) of the SHARE cohort, including 27 countries (Austria, Belgium, Bulgaria, Croatia, Cyprus, Czech Republic, Denmark, Estonia, Finland, France, Germany, Greece, Hungary, Italy, Latvia, Lithuania, Luxembourg, Malta, Netherlands, Poland, Portugal, Romania, Slovakia, Slovenia, Spain, Sweden, and Switzerland) were used for this analysis. All study participants provided informed consent, and the study received ethical approval from the Ethics Council of the Max Planck Society and the relevant national ethics committees. The analysis included 38,911 women and 29,136 men (Table 1).

**Table 1:** Weighted distributions of participant characteristics among adults aged 50 years and older (*n* = 68,047) from 27 European countries participating in the Survey of Health, Ageing and Retirement in Europe (SHARE), 2021–2022.

Characteristic	Frequency (weighted percentage)	
	Women (*n* = 38,911)	Men (*n* = 29,136)
*Age (years)*		
50–64	13,243 (50.8)	9429 (49.2)
65–79	18,835 (54.6)	15,148 (45.4)
80+	6833 (63.1)	4559 (36.9)

*Employment status*		
Employed or self-employed	7683 (29.1)	6624 (38.6)
Unemployed	660 (2.4)	591 (4.3)
Retired	24,150 (48.2)	20,421 (50.2)
Not working due to permanent sickness or disability	929 (3.4)	807 (4.0)
Homemaker	4220 (13.3)	117 (0.8)
Other	695 (2.1)	237 (0.9)

*Residence*		
Home	38,430 (98.9)	28,877 (99.2)
Nursing home	420 (0.8)	209 (0.5)

*Self-reported health*		
Excellent	2009 (5.5)	1714 (6.8)
Very good	6084 (16.9)	4771 (18.9)
Good	15,079 (39.9)	11,627 (40.6)
Fair	11,438 (27.3)	8078 (24.8)
Poor	4216 (10.2)	2856 (8.4)

*Body mass index (kg/m*^*2*^*)*		
<18.5	691 (2.2)	162 (0.6)
18.5–24.9	13,684 (39.4)	8308 (30.2)
25.0–29.9	13,762 (33.9)	13,236 (45.9)
30.0–34.9	6653 (14.6)	5210 (16.4)
35.0–39.9	2018 (4.4)	1177 (3.4)
>40.0	679 (1.6)	321 (0.9)

*Smoking status*		
Non-smoker	33,981 (86.0)	23,815 (79.1)
Current smoker	4847 (13.8)	5223 (20.4)

*Moderate physical activity*		
>1 time per week	24,678 (59.3)	19,344 (64.3)
1 time per week	5289 (14.9)	3936 (15.0)
1–3 times per month	2706 (8.0)	2073 (7.8)
Hardly ever or never	6149 (17.6)	3701 (12.6)

*Vigorous physical activity*		
>1 time per week	10,589 (28.0)	10,446 (38.2)
1 time per week	5320 (12.6)	4001 (12.8)
1–3 times per month	4198 (9.4)	3239 (10.1)
Hardly ever or never	18,705 (49.8)	11,354 (38.5)

### 2.2 Measures

#### 2.2.1 Hypertension diagnosis

Participants were asked the following question to identify current/previous diagnosis of hypertension: “Has a doctor ever told you that you had/Do you currently have any of the conditions on this card? With this we mean that a doctor has told you that you have this condition, and that you are either currently being treated for or bothered by this condition.” Hypertension was listed as one of the possible responses.

### 2.3 Statistical analysis

Sample weights (person-level analysis) were applied to calculate gender-specific weighted prevalence and 95% confidence intervals (CI) of current/previous hypertension diagnosis. Prevalence estimates were calculated for ages of 50–64, 65–79, and 80+ years. Subgroup analyses were carried out based on European regions following the EuroVoc classification, including: Central and Eastern Europe (Bulgaria, Croatia, Czech Republic, Hungary, Poland, Romania, Slovakia, and Slovenia), Northern Europe (Denmark, Estonia, Finland, Latvia, Lithuania, and Sweden), Southern Europe (Cyprus, Greece, Italy, Malta, Portugal, and Spain), and Western Europe (Austria, Belgium, France, Germany, Luxembourg, Netherlands, and Switzerland (EuroVoc, 2014). Statistical analyses were performed using SPSS v.30 (IBM Corp., Chicago, IL, USA).

## 3 Results

### 3.1 Overall prevalence

Among individuals aged 50–64 years, men had a higher prevalence of current/previous hypertension diagnosis (39.4% vs. 33.5%; Table 2). In the 65–79 age group, prevalence was comparable between genders (women: 63.1%, men: 62.6%). In the 80+ age group, women had higher prevalence (78.0% vs. 71.8%).

**Table 2:** Weighted prevalence of self-reported current/previous hypertension diagnosis among women (*n* = 38,911) and men (*n* = 29,136) aged 50 years and older from 27 European countries in the Survey of Health, Ageing and Retirement in Europe (SHARE), 2021–2022.

Region and age group	*n*	Current/previous hypertension diagnosis (%)
Women		
Europe		
50–64 years	13,243	33.5 (32.8, 34.2)
65–79 years	18,835	63.1 (62.3, 64.0)
80+ years	6833	78.0 (77.0, 79.0)

Central and Eastern Europe		
50–64 years	4653	44.1 (42.4, 45.8)
65–79 years	6340	73.4 (71.9, 75.0)
80+ years	1840	85.2 (83.1, 87.3)

Northern Europe		
50–64 years	2551	33.1 (30.1, 36.1)
65–79 years	3913	56.2 (52.9, 59.5)
80+ years	1719	75.1 (70.6, 79.6)

Southern Europe		
50–64 years	2173	30.4 (29.2, 31.6)
65–79 years	3329	69.9 (68.4, 71.5)
80+ years	1327	85.5 (84.0, 87.1)

Western Europe		
50–64 years	3866	31.2 (30.2, 32.2)
65–79 years	5253	54.3 (53.0, 55.6)
80+ years	1947	70.2 (68.5, 71.8)

Men		
Europe		
50–64 years	9429	39.4 (38.7, 40.1)
65–79 years	15,148	62.6 (61.7, 63.6)
80+ years	4559	71.8 (70.3, 73.2)

Central and Eastern Europe		
50–64 years	3235	39.7 (38.0, 41.4)
65–79 years	5024	64.6 (62.7, 66.6)
80+ years	1124	75.2 (71.5, 78.9)

Northern Europe		
50–64 years	1961	37.5 (34.3, 40.6)
65–79 years	2867	57.0 (53.4, 60.6)
80+ years	9,76	70.2 (63.9, 76.4)

Southern Europe		
50–64 years	1352	43.1 (41.8, 44.4)
65–79 years	2774	74.2 (72.6, 75.8)
80+ years	1044	80.5 (78.3, 82.7)

Western Europe		
50–64 years	2881	36.8 (35.7, 37.9)
65–79 years	4483	55.8 (54.4, 57.3)
80+ years	1415	64.6 (62.3, 66.8)

Data are presented as prevalence (%) and 95% confidence interval. Regions are classified as: Central and Eastern Europe (Bulgaria, Croatia, Czech Republic, Hungary, Poland, Romania, Slovakia, and Slovenia), Northern Europe (Denmark, Estonia, Finland, Latvia, Lithuania, and Sweden), Southern Europe (Cyprus, Greece, Italy, Malta, Portugal, and Spain), and Western Europe (Austria, Belgium, France, Germany, Luxembourg, Netherlands, and Switzerland)

### 3.2 Gender differences by region

In Central and Eastern Europe, women had higher prevalence across all age groups (44.1–85.2% vs. 39.7–75.2%). In Northern Europe, prevalence rates were similar between genders at all age groups (women: 33.1–75.1%, men: 37.5–70.2%). In Southern Europe, men had higher prevalence in the 50–64 age group (43.1% vs. 30.4%) and 65–79 age group (74.2% vs. 69.9%), while women had higher prevalence in the 80+ age group (85.5% vs. 80.5%). In Western Europe, men had higher prevalence in the 50–64 age group (36.8% vs. 31.2%), similar prevalence was in the 65–79 age group (women: 54.3%, men: 55.8%), while women had higher prevalence in the 80+ age group (70.2% vs. 64.6%).

### 3.3 Regional variation among women

Women aged 50–64 years had a higher prevalence in Central and Eastern Europe (44.1%), compared to all three other regions (30.4–33.1%). At 65–79 years, prevalence was higher in Central and Eastern Europe (73.4%) compared to all three other regions (54.3–69.9%). In the same age group, prevalence was higher in Southern Europe (69.9%), compared to Northern (56.2%) and Western Europe (54.3%). At 80+ years, prevalence was higher in Central and Eastern Europe (85.2%) and Southern Europe (85.5%) compared to Northern (75.1%) and Western Europe (70.2%).

### 3.4 Regional variation among men

Men aged 50–64 years had a higher prevalence in Southern Europe (43.1%) compared to all three other regions (36.8–39.7%). In the same age group, prevalence was higher in Central and Eastern Europe (39.7%) compared to Western Europe (36.8%). At 65–79 years, prevalence was higher in Southern Europe (74.2%) compared to all three other regions (55.8–64.6%). In the same age group, prevalence was higher in Central and Eastern Europe (64.6%), compared to Northern (57.0%) and Western Europe (55.8%). At 80+ years, prevalence was higher in Southern Europe (80.5%) compared to Northern (70.2%) and Western Europe (64.6%). In the same age group, prevalence was also higher in Central and Eastern Europe (75.2%) compared to Western Europe (64.6%).

## 4 Discussion

Among adults aged 50–64 years, the prevalence of current or previous hypertension diagnosis was higher in men; it was similar between genders at ages 65–79, while among those aged 80+ years, it was higher in women. However, this pattern varied across European regions. The highest prevalence of current/previous hypertension diagnosis was generally found in Southern and Central/Eastern Europe compared to Northern and Western regions.

Current/previous hypertension diagnosis increased with age, reaching the highest values in the 80+ years group. Similar age patterns were observed in other studies. For example, in the US, hypertension prevalence also increases with age, from 22.4% (18–39 years) to 54.5% (40–59 years), and reaches even 74.5% (60+ years) (Ostchega et al., 2020). In China, hypertension prevalence also increased with age, from 4.0% in the 18–24 years group to 60.2% in the ≥75 years group (Wang et al., 2018).

The current study shows that the gender difference may be age-dependent as the prevalence of current/previous hypertension diagnosis is higher in men, but only at 50–64 years. With advancing age, the difference is diminished, and trends change in adults aged 80+ years, where hypertension prevalence is higher in women. An elevated blood pressure may occur because of increased sympathetic and renin-angiotensin activity that causes vasoconstriction and sodium retention, shifting pressure regulation (Reckelhoff, 2023). Sex steroids can affect these mechanisms thus potentially explaining some of the gender differences in hypertension (Reckelhoff, 2023). Additionally, with menopause there are associated changes in arterial stiffness and central wave reflection, which may also potentially contribute to some of the differences between genders in hypertension prevalence (Samargandy et al., 2020; Mancia et al., 2023).

Men and women from Central/Eastern Europe and Southern Europe have a higher prevalence of hypertension diagnosis compared to other regions. Similar findings according to different regions were found in a recent pooled analysis examining the worldwide prevalence of hypertension (NCD Risk Factor Collaboration (NCD-RisC), 2021). The gender and age difference in prevalence of diagnosis also varied within a region. These patterns may reflect broader systemic differences in healthcare infrastructure, public health policy, and population health profiles across the European regions (Schwettmann et al., 2023). For example, data indicate that the prevalence of low physical activity is higher in Central/Eastern Europe and Southern Europe compared to Northern and Western Europe (Nikitara et al., 2021). Tobacco use is another factor that may help explain these differences, as smoking rates tend to be higher in Eastern and Southern European countries compared with those in Northern and Western Europe (Feliu et al., 2019). These differences coincide with lower socioeconomic status in these former regions, which is associated with higher blood pressure (Mackenbach et al., 2008; Leng et al., 2015). Importantly, the observed disparities suggest that public health initiatives aimed at reducing hypertension prevalence may need to be region-specific. Tailoring prevention and management strategies to local socioeconomic, behavioural, and health-system contexts could improve equity and the overall effectiveness of hypertension control across Europe.

The strengths of the study include the large sample, the number of countries represented, and the use of recently collected data (2021–2022). However, a limitation is the self-report of hypertension diagnosis. Previous studies on the validity of self-reported hypertension showed varying degrees of accuracy compared to objectively measured methods (Taylor et al., 2010). Additionally, the survey examined current or previous hypertension diagnosis but did not differentiate between the two. Finally, while 27 countries were included in the analysis, not all European nations were represented. For instance, only three former Soviet states (Estonia, Latvia, and Lithuania) were included.

## 5 Conclusion

In conclusion, among adults aged 50–64 years, the prevalence of current or previous hypertension diagnosis was higher in men; it was similar between genders at ages 65–79, while among those aged 80 and older, it was higher in women, although this pattern varied across European regions. The prevalence rates of current/previous hypertension diagnosis in the same gender and age group were generally higher in Central/Eastern Europe and Southern Europe compared to Northern and Western regions.

## CRediT authorship contribution statement

**Jozo Grgic:** Writing – review & editing, Writing – original draft, Methodology, Investigation, Formal analysis, Conceptualization. **Vanessa Kristina Wazny:** Writing – review & editing, Writing – original draft, Investigation, Conceptualization.

## Declaration of competing interest

The authors declare that they have no known competing financial interests or personal relationships that could have appeared to influence the work reported in this paper.

## Data Availability

Data used for this study are available upon request from the Survey of Health, Ageing and Retirement in Europe (SHARE) website: https://share-eric.eu/
